# Perinatal risk factors for early onset of Type 1 diabetes in a 2000–2005 birth cohort

**DOI:** 10.1111/j.1464-5491.2009.02878.x

**Published:** 2009-12

**Authors:** C S Algert, A McElduff, J M Morris, C L Roberts

**Affiliations:** * Kolling Institute of Medical Research, University of SydneySydney; † Department of Obstetrics and Gynaecology, Royal North Shore HospitalSt Leonards, Australia; ‡ Department of Endocrinology, Royal North Shore HospitalSt Leonards, Australia

**Keywords:** birthweight, Caesarean section, pregnancy, record linkage, Type 1 diabetes

## Abstract

**Aims:**

To examine perinatal risk factors for the onset of Type 1 diabetes before 6 years of age, in a 2000–2005 Australian birth cohort.

**Methods:**

Data from longitudinally linked delivery and hospital admission records (until June 2007) were analysed. Diabetes in mothers and children was identified from International Classification of Diseases 10 diagnosis codes in the hospital records.

**Results:**

There were 272 children admitted to hospital with a first diagnosis of diabetes out of 502 040 live births. Incidence for the infants born in 2000 was 16.0 per 100 000 person-years. Maternal Type 1 diabetes was a significant risk factor [crude relative risk (RR) 6.33], but maternal Type 2 diabetes and gestational diabetes were not significantly associated with diabetes in the child. Late preterm birth (34–36 weeks) (RR 1.64) and caesarean section (RR 1.30) increased the risk of a diabetes admission. Size-for-gestational-age was significantly associated with onset of diabetes (small-for-gestational age RR 0.48), but neither birth weight categories nor birth weight as a continuous variable were associated with risk of diabetes. Increasing maternal age was associated with an increased risk of diabetes in the child (RR 1.13 for each additional 5 years of age).

**Conclusions:**

This study identified risk factors associated with onset of Type 1 diabetes before 6 years of age, in a recent birth cohort. Size-for-gestational-age had a consistent association with risk of early onset of Type 1 diabetes, small size being protective. Size-for-gestational-age measures should be preferred to birth weight thresholds when assessing risk of diabetes.

## Introduction

The incidence of childhood-onset Type 1 diabetes is rising in developed countries [1,2]. This rising incidence is likely to be a result of changes in environmental risk factors, some from the perinatal period, that can initiate or accelerate the development of the disorder [3]. Previous studies of perinatal risk factors for childhood diabetes have relied upon births from earlier decades. We used linked population health data to examine perinatal risk factors for the onset of Type 1 diabetes in early childhood (< 6 years of age), in children born during 2000–2005.

## Methods

Longitudinally linked de-identified data from a database of all deliveries (Midwives Data Collection) and from administrative data of all hospital admissions in the state (Admitted Patient Data Collection) of New South Wales (NSW), Australia were analysed. These databases have been described previously [4]. All singleton live births from January 2000 until December 2005 were included, with linkage to childhood hospital admission records available up to 6 years of age or before 1 July 2007, whichever was earlier. Multiple gestations were excluded due to their unique perinatal risks and small numbers. If an infant’s family moved out of NSW, that infant would have been lost to follow-up at that point. International Classification of Diseases 10 diagnosis codes on hospital discharge records were used to identify children’s diagnoses and maternal diabetes, which have been shown to be well reported.[5]

Maternal and pregnancy factors that were available on the databases included maternal diabetes, age, parity, preeclampsia, gestational age, caesarean section delivery, infant sex and birth weight. Information on paternal diabetes status was not available. Size-for-gestational-age was determined using standard birth weight percentile charts [6]. Small-for-gestational-age (SGA) and large-for-gestational-age (LGA) were defined as < 10th percentile and > 90th percentile birth weight for gestational age, respectively, conventional in obstetric and neonatal studies. Preterm birth was categorized as < 34 weeks and 34–36 weeks (late preterm) [7].

Data on hospital admissions were only available until June 2007, so infants in the cohort born after June 2001 were censored before reaching 6 years old. This resulted in a greater proportion of early diagnoses. To examine whether this could affect the results, we separately analysed infants whose first diagnosis was before 3 years with infants whose first diagnosis was at ≥ 3 years old. The effects, by age at diagnosis, were compared using a chi-square test of homogeneity across subgroups [8]. *P* < 0.05 for this test would be evidence that homogeneity cannot be assumed and that the effects differ by subgroup.

Crude relative risks (RR) and 95% confidence intervals (CI) were calculated for the available risk factors. To adjust for multiple risk factors and for person-years in the study, Cox proportional hazard models were used to calculate hazard ratios (HR) and their CIs. The HR is equivalent to a RR for an uncommon condition such as Type 1 diabetes. Parsimonious models were selected, where only factors with a *P* < 0.10 level of statistical significance were retained for the final model.

## Results

There were 272 children admitted with a diabetes mellitus diagnosis out of 502 040 singleton infants. Of the infants born during the first 12 months of the study period, 81 had an admission for diabetes (16.0/100 000 person-years) before reaching 6 years of age, with a median age at first admission of 3.3 years. For all births, the median age at first admission with a diabetes diagnosis was 2.9 years. Only 12 children (4.4%) were diagnosed before 1 year old.

The frequencies and crude RRs for maternal and pregnancy risk factors are shown in Table 1. Maternal Type 1 diabetes was a strong risk factor for onset of Type 1 diabetes in the child (RR 6.30), but with a wide CI, based upon five cases. Gestational diabetes had a point estimate of risk greater than unity (RR 1.20), but this did not approach the level of statistical significance (*P* = 0.50). There were no cases among infants whose mothers had Type 2 diabetes. SGA infants had a reduced risk of diabetes (RR 0.48), whereas infants delivered by caesarean had an increased risk (RR 1.30). Infants born at 34–36 weeks had the highest risk by gestational age, while early preterm (< 34 weeks) infants showed no evidence of increased risk. Maternal age and gestational age as continuous variables were significantly associated with diabetes onset using univariate Cox regression. The risk of diabetes in the child increased by 13% (RR 1.13; 95% CI 1.01, 1.26) for every 5 years of maternal age, and the risk of diabetes was decreased by 12% (RR 0.88; 95% CI 0.82, 0.95) with each additional week of gestational age after 34 weeks. Birth weight as a continuous variable was not significantly associated with diabetes (RR 1.09; 95% CI 0.97, 1.22 for each additional 500 g), but size-for-gestational-age category, treated as an interval variable (SGA, 10–90th, LGA), was strongly associated with onset of diabetes (*P* = 0.002).

**Table 1:** Crude relative risks for admission to hospital with diabetes in children aged ≤ 5 years

Risk factor	Infants with diabetes/*N*	Crude relative risk RR (95% CI)
Maternal diabetes		
Type 1 diabetes	5/1486	6.33 (2.62, 15.3)
Type 2 or unspecified diabetes mellitus	0/1800	0.0 (0.0, 8.4)*
Gestational diabetes	17/26 671	1.20 (0.73, 1.96)
Diabetes of unknown type	1/3750	0.50 (0.07, 3.57)
No maternal diabetes	249/468 333	1.0 (referent)
Maternal age, years		
≥ 40	12/16 252	1.46 (0.81, 2.65)
30–39	138/243 842	1.12 (0.88, 1.44)
< 30	122/241 770	1.0 (referent)
Nulliparous mother	112/209 603	0.98 (0.77, 1.24)
Preeclampsia†	13/15 683	1.56 (0.89, 2.72)
Gestational age, weeks		
24–33	3/6749	0.76 (0.24, 2.37)
34–36	19/19 711	1.64 (1.01, 2.65)
37–39	130/221 151	1.0 (referent)
≥ 40	120/254 429	0.80 (0.63, 1.03)
Caesarean section delivery	82/124 772	1.30 (1.01, 1.69)
Planned caesarean section	46/69 531	1.31 (0.95, 1.81)
Caesarean section after labour	36/55 241	1.29 (0.91, 1.85)
Male infant	141/258 714	1.01 (0.80, 1.28)
Birth weight < 2500 g	9/22 227	0.74 (0.38, 1.44)
Birth weight 2500–3999 g	229/417 675	1.0 (referent)
Birth weight ≥ 4000 g	34/61 993	1.00 (0.70, 1.43)
Birth weight percentile for gestational age		
SGA (< 10th)	13/48 670	0.48 (0.28, 0.84)
10–90th	221/401 327	1.0 (referent)
LGA (> 90th)	38/52 043	1.32 (0.94, 1.86)

LGA, large-for-gestational-age; SGA, small-for-gestational-age; RR, relative risk.

* Confidence interval calculated by replacing the zero cell with a value of 0.5.

† Includes eclampsia.

The follow-up censoring date of 30 June 2007 meant that 74.4% of children had at least 3 years of follow-up time, including 25.0% of children who had full follow-up until their sixth birthday. Table 2 shows the comparison, for selected risk factors, between children diagnosed at < 3 years vs. ≥ 3 years old. Both preeclampsia and caesarean were statistically significant risk factors for a diabetes diagnosis before 3 years. The two factors were not unrelated, as maternal preeclampsia had a risk ratio for delivery by caesarean section of RR = 1.79 (95% CI 1.76, 1.83). The size-at-birth indicators were not risk factors for diagnosis before 3 years of age, but both SGA (reduced risk) and LGA (increased risk) were associated with first admission diabetes at ≥ 3 years. However, all chi-square tests for homogeneity by age at diagnosis were *P* ≥ 0.05, so there was no statistical evidence that any of the risk factor effects were significantly different by age at diagnosis.

**Table 2:** Crude relative risks for first admission with diabetes < 3 years vs. ≥ 3 years of age

Risk factor	First admission at < 3 years RR (95% CI) *N* = 141	First admission at 3–5 years RR (95% CI) *N* = 131	χ^2^ test of homogeneity of RRs by age at diagnosis*
Maternal diabetes			
Type 1 diabetes	7.51 (2.39, 23.6)	5.12 (1.27, 20.8)	*P* = 0.68
Gestational diabetes	1.53 (0.83, 2.84)	0.87 (0.38, 1.97)	*P* = 0.28
No maternal diabetes	1.0 (referent)	1.0 (referent)	
Maternal age, years			
≥ 40	2.35 (1.16, 4.74)	0.72 (0.23, 2.29)	*P* = 0.09
30–39	1.30 (0.92, 1.84)	1.00 (0.71, 1.41)	*P* = 0.29
< 30	1.0 (referent)	1.0 (referent)	
Preeclampsia†	2.03 (1.03, 3.98)	0.95 (0.35, 2.58)	*P* = 0.19
Gestational age, weeks			
34–36	1.76 (0.93, 3.33)	1.48 (0.71, 3.10)	*P* = 0.73
37–39	1.0 (referent)	1.0 (referent)	
≥ 40	0.72 (0.51, 1.02)	0.87 (0.61, 1.24)	*P* = 0.45
Caesarean section	1.46 (1.03, 2.08)	1.21 (0.82, 1.77)	*P* = 0.47
Birth weight percentile for gestational age			
SGA (< 10th)	0.64 (0.32, 1.26)	0.31 (0.11, 0.85)	*P* = 0.24
10–90th	1.0 (referent)	1.0 (referent)	
LGA (> 90th)	1.06 (0.63, 1.79)	1.62 (1.02, 2.57)	*P* = 0.23

LGA, large-for-gestational-age; SGA, small-for-gestational-age; RR, relative risk.

* *P* ≥ 0.05 indicates no evidence of a statistically significant difference in effect by age at first diagnosis category.

† Iincludes eclampsia.

Because of the possibility that risks could be different by age at first diagnosis, separate multivariable models were performed for children diagnosed at < 3 years and ≥ 3 years. In the final multivariable model for diagnosis at < 3 years, only maternal Type 1 diabetes (HR 6.72, 95% CI 2.13, 21.2), maternal age ≥ 40 years (HR 2.03, 95% CI 1.03, 3.98) and preeclampsia (HR 2.00, 95% CI 1.02, 3.94) remained statistically significant risks. Caesarean section (*P* = 0.10) and preterm birth (*P* = 0.12) were close to being retained in this model. In the final multivariable model for diagnosis at ≥ 3 years, maternal Type 1 diabetes (HR 4.34, 95% CI 1.07, 17.7) and SGA (HR 0.32, 95% CI 0.12, 0.85) were significantly associated with diabetes, but LGA was not (HR 1.57, 95% CI 0.99, 2.49).

## Discussion

Onset of diabetes before 6 years old was associated with maternal Type 1 diabetes, but not gestational diabetes or Type 2 diabetes. Other population cohort studies have found a risk from maternal diabetes, but were not able to distinguish fully by type [9–11]. The CI around the estimated RR for gestational diabetes was wide and does not preclude a moderately increased risk of diabetes in the child. Much larger study population numbers, such as in a systematic review, would be required to identify such a risk confidently.

Size-for-gestational-age had consistent associations with the risk of admission for diabetes, particularly for first diagnoses after 3 years of age. In that subgroup, SGA was protective (HR = 0.32) and LGA bordered on an increased risk compared with 10–90th percentile birth weight infants. Our results illustrate the importance of using size-for-gestational-age as an exposure factor in preference to birth weight alone, which confounds growth and maturity. A threshold of 4000 g was the 83rd percentile birth weight for male infants at 40 weeks, for example, but was the 97th percentile at 37 weeks and would be LGA. Using birth weight alone will introduce additional random variation into comparisons based on infant size, and would usually result in underestimation of any relative risk associated with size. Of two previous population studies that looked at size-for-gestational age, both reported higher rates of Type 1 diabetes for children who had been ≥ 80th percentile than for children with birth weight ≥ 4000 g [10,12].

Preeclampsia was significantly associated with childhood diabetes in our study, but only among children diagnosed before 3 years of age. Preeclampsia as a risk factor has been reported by some [3,13] but not all studies [9,10] in which diagnoses up to 15 years of age were recorded. Caesarean section was a crude risk factor for diabetes, although it did not remain statistically significant in the adjusted analyses. The crude risk associated with caesarean section in our study was similar to the results of a meta-analysis that reported a pooled odds ratio of 1.23 (95% CI 1.15, 1.32) for caesarean section [14]. Caesarean section is an important risk factor since it is a common exposure (24.9% of deliveries in this study) and rates of caesarean section are rising in developed countries. One of the possible causal pathways hypothesized is that infants delivered by caesarean section do not have the same exposure to maternal bacteria as infants delivered vaginally, as evidenced by differences in gut microbiotic compositions [15].

Preterm birth before 34 weeks showed no evidence of an association with early onset of diabetes in our study, but late preterm (34–36 weeks) did. Two other studies have looked at subcategories of preterm birth and did not find a peak at 34–36 weeks [16,17]. Another study reported a linear decrease in risk of diabetes with increasing gestational age, but only for males [18]. Those studies included initial diagnoses up until 15 years old. It is possible that preterm birth could be associated with earlier onset of Type 1 diabetes. A recent study of HLA genotypes found that high-risk genotypes were associated with shorter gestations [19].

Identification of cases for this study depended upon children having a hospital admission if they developed Type 1 diabetes, whereas previous population studies have generally utilized diabetes registries for case ascertainment. An earlier NSW study that drew upon a children’s diabetes register reported that the mean incidence of diabetes in children aged 0–4 years during the period 1997–2002 was 13.6 per 100 000 person-years [2]. The incidence in our study for children 0–5 years old was higher (16.0/100 000 person-years), reflecting the additional year for a potential diagnosis, and also perhaps a continuation of the rising trend of childhood diabetes. The comparability of these incidence rates supports the use of hospital admission records as a reliable method to identify cases of Type 1 diabetes in populations.

In conclusion, the timing and magnitude of risk factors can help elucidate the aetiology of Type 1 diabetes, although this knowledge may not offer good prospects for effective intervention in the short term. If preeclampsia or caesarean section risk are causally linked to development of diabetes, that could offer hope that altered pregnancy management might effect change in the incidence of Type 1 diabetes. Also interesting is that SGA infants appear to have a reduced risk of Type 1 diabetes. SGA is used as a proxy for intrauterine growth retardation and a marker of a poor neonatal outcome, but there may be some aspects of the adaptation of these infants to their *in utero* environment that are protective.

## Abbreviations

CI: confidence interval
HR: hazard ratio
LGA: large-for-gestational-age
NSW: New South Wales
RR: relative risk
SGA: small-for-gestational-age
